# Investigating soil properties and vegetation parameters in different biochar-amended vegetated soil at large suction for application in bioengineered structures

**DOI:** 10.1038/s41598-022-22149-5

**Published:** 2022-12-08

**Authors:** Rojimul Hussain, K. Ravi

**Affiliations:** Department of Civil Engineering, Indian Institute of Technology, Guwahati, Guwahati, India

**Keywords:** Engineering, Civil engineering

## Abstract

Bioengineered structures, such as landfill cover, vegetated slopes or embankments, green roof and turf are comprised of soil and vegetation where vegetation imparts stability and protection through root reinforcement and hydrologic action. Soil in bioengineered structures often compacted and subjected to prolong drying due to irregular irrigation which necessitates the investigation of soil properties and vegetation growth in biochar-amended soil (BAS) under large suction range and it is scarce in the literature. In the present study, the effect of different biochar types on soil properties, and the vegetation growth in compacted soil and under large suction range was investigated for application in bioengineered structures. The results revealed that the biochar amendment decreased the dry density (5–32%) and increased the water retention capacity (*θ*_*s*_ by 15–104%, *θ*_*1500*_ by 82–445% and plant available water content (PAWC) by 22–55%), pH (28–77%) and cation exchange capacity (CEC, 16–723%) of the soil. Further, the vegetation growth i.e., vegetation density, dry root mass and shoot mass increased by 8–13%, 33–108% and 4–157% respectively after biochar amendment. The vegetation wilting was started at a higher suction (~ 900 kPa) relative to bare soil (800 kPa), the permanent wilting point (PWP) increased (by 3–35%) and the complete photosynthetic activity remained unchanged at a higher suction (1600 kPa) relative to bare soil (1050 kPa) after biochar amendment. Among the biochar types i.e., Sugarcane Bagasse biochar (SBB), Mesquite biochar (MB) and Water Hyacinth biochar (WHB) tested, the MB showed the best performance i.e., the suitable vegetation growth and health status. The improved water retention due to increased porosity, specific surface area (SSA) and presence of hydrophilic functional groups, and the higher pH, CEC and lower dry density in BAS attributed to the higher vegetation growth. The findings of the present study suggest the application of BAS in bioengineered structures.

## Introduction

**Figure 1:**
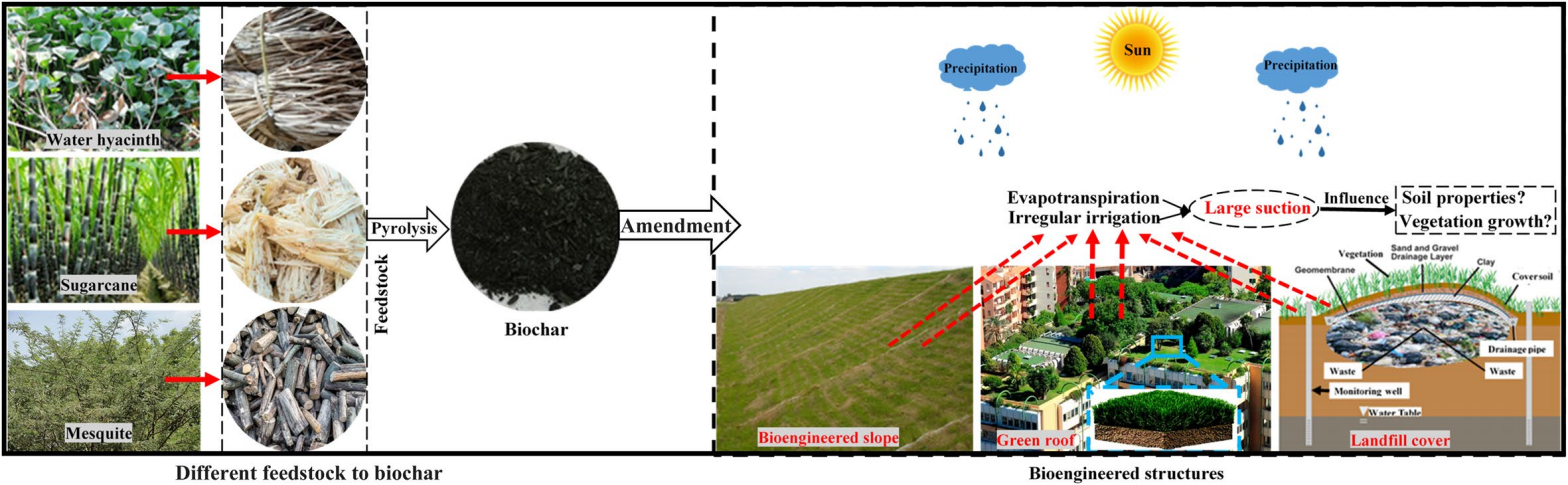
Portraying the production of biochar from different feedstock and amending them in bioengineered structures.

Bioengineered or Geotechnical structures, such as landfill cover, vegetated road, railway and hydraulic embankments or slopes, green roof and turf, as shown in Fig. 1 are comprised of soil and vegetation where soil is often compacted for achieving the structural stability^1,2^. The vegetation in these structures plays significant role in stabilizing and protecting the structures from surface erosion and failure^3,4^. The vegetation roots act as soil reinforcement and anchor the soil around and hence increases the strength and stability^5,6^. Further, the root water uptake to meet the plants need and compensate the water loss due to evapotranspiration generates suction in the root zone or soil which in turn contributes to the strength and stability^7,8^. Therefore, proper growth or development along with healthy status of vegetation is crucial for effective functioning or stability of the bioengineered structures. The plant health could be assessed from some parameters such as stomatal conductance (SC) and photosynthesis yield (PY) which are also interrelated to the evapotranspiration induced suction and the stability^9^. The SC is the rate at which CO_2_ enter and water vapour exit through the stomata in plants. The rate of passes of the water vapour is the transpiration rate and is related to the root water uptake^10^. The PY is the fraction of light energy (in terms of quantum) converted to chemical energy during photosynthesis process in plants i.e., indirectly represents the rate of photosynthesis and hence the growth of the vegetation^11,12^.

The growth and health of vegetation predominantly depend on the soil properties, especially water retention characteristics and physicochemical properties i.e., soil with suitable hydraulic and physicochemical properties assists the better development of vegetation^13,14^. The amendment of biochar in soil is reported to improve the soil properties^12,15,16^. Biochar is a microbial non-degradable carbonaceous product obtained after pyrolysis of biomass under limited oxygen conditions^17^. The pyrolysis of biomass into biochar and amending them in soil is a sustainable way of controlling the release of atmospheric CO_2_, managing the waste biomass and sequestering carbon for longer duration^18^. Further, biochar amendment in soil could also improve the microbial activity and capture the organic and inorganic (heavy metals) pollutants^19–23^. These positive attributes of biochar are due to the porous structure and presence of surface functional groups in biochar which are controlled by the types of feedstock and pyrolysis temperature^24,25^.

The effect of biochar on soil properties of relatively loose agricultural soil was extensively studied by the past researchers^26–31^. Few studies were also conducted on compacted BAS mimicking the bioengineered structures^12,32–36^. Reddy et al.^15^ documented the improved physicochemical properties of silty clay soil amended with wood pellet biochar. Chen et al.^37^ reported a higher water retention capacity of sandy soil amended with wheat straw biochar. Similarly, Hussain et al.^16^ reported an increased water retention and decreased infiltration rate of silty sand after amendment of biochar produced from mesquite and water hyacinth. Ni et al.^38^ investigated the hydraulic properties of biochar-amended vegetated soil and reported the improved soil hydraulic properties and vegetation growth due to biochar amendment. Recently, Ng et al.^36^ tried to establish the relation between the soil suction and plant (*Schefflera arboricola*) parameters in compacted biochar-amended completely decomposed granite soil. However, the suction measured was very small (< 90 kPa) for field application such as bioengineered structures where soil subjects to prolong drying (drought) due to rare irrigation or low maintenance. The drought stress severely affects the growth and development of vegetation^39^. Further, at this lower (< 90 kPa) suction, most of the soils (especially, fine-grained soil) remain in saturated state and the plant parameters i.e., SC, PY and root or shoot mass may not change, since these parameters change with drought or increased suction. Therefore, the investigation of soil properties and vegetation growth and health parameters in soil amended with biochar produced from diverse feedstock, and considering the large suction range or drought stress is needed for application in bioengineered structures. The objective of the present study is to investigate the soil and plant parameters in compacted different types biochar-amended soil at relatively large suction for potential application in bioengineered structures. A non-crop species, *Axonopus compressus* (grass) was grown in compacted soil columns prepared with 5% and 10% (w/w) biochar produced from biomass of mesquite, water hyacinth and sugarcane bagasse, thereafter the soil and plant parameters were measured and reported.

## Materials and methods

### Biochar

Biochar in the present study was produced from the feedstock of sugarcane bagasse (SB), water hyacinth (WH) and mesquite biomass. The feedstock was slow pyrolysed at 400 °C for a period of 45 min using a fixed bed reactor pyrolyser. Mesquite and water hyacinth are the fast-growing invasive weed and the SB is the waste by-product of the sugar industries, these are abundantly available for pyrolysis^40^. Therefore, the production of biochar from these feedstocks could at least be the management of the waste. The biochar produced are characterised for physicochemical and microstructural properties described next. The physicochemical properties, such as elemental composition, ash content (AC), volatile matter (VM), cation exchange capacity (CEC), pH and electrical conductivity (EC) of the biochar were determined according to the procedure described in Hussain et al.^35^. Similarly, the microstructural properties, such as functional groups, mineral composition or crystallinity, surface morphology, specific surface area (SSA) were evaluated by FTIR, XRD, FESEM and BET analysis as described in Hussain et al.^16^.

### Soil

Soil was collected from inside the campus of Indian Institute of Technology (IIT) Guwahati India (26.19°N, 91.69°E). It is majorly used for construction activity i.e., the construction of embankment or slopes in the region. The soil collected from the site was oven-dried and sieved through 4.75 mm sieve for further analysis. The physicochemical properties of the soil, such as grain size distribution (GSD), specific gravity, consistency limits, dry density, pH, EC, CEC and SSA were determined according to the procedure outlined by ASTM standard^41–46^.

### Plant species

To see the effect of biochar amendment on the vegetation growth, a non-crop species, broadleaf carpet grass (*Axonopus compressus*) was grown. Permission was obtained from relevant authority for collecting the Axonopus compressus plants. It is a perennial grass with fibrous root system and spreads by creeping stolon and underground rhizomes. The abundant availability, high root tensile strength (51–60 MPa), drought tolerance characteristics, and the land rehabilitation and reforestation ability of the grass make its regular use in the slopes stabilization or erosion control in South–East Asia^47–50^. The roots mainly grow within the top 40 cm soil depth. Further, as a non-crop species, it requires less maintenance in terms of nutrient supplement and water irrigation, thus suitable for bioengineered structures^9^.

### Test procedure

#### Transplantation of the plant and establishment of SWRC

**Figure 2:**
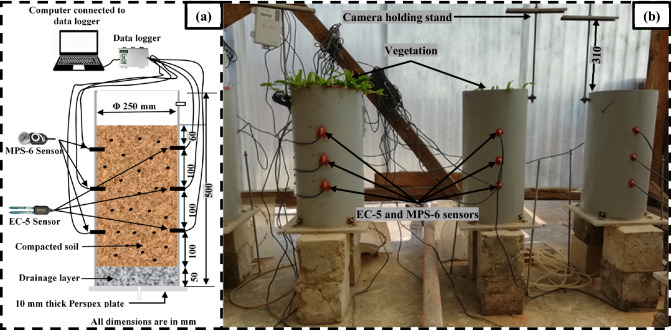
(**a**) schematic diagram (**b**) real image under transparent house of the experimental setup adopted for growing the vegetation.

The test setup used for growing the vegetation is shown in Fig. 2a,b. The preparation of soil column of dimension 250 mm diameter and 500 mm length and the installation of sensors for monitoring suction and moisture content were described elsewhere in Hussain and Ravi^51^. The columns were prepared with 5% and 10% (w/w) biochar of different types and selected based on the preliminary investigations conducted for the biochar types considered. Three independent replicates of column for each soil and biochar combination were prepared. The amendment of biochar higher than 10% led to the accumulation of the biochar during mixing i.e., non-uniform mix and decreased the soil dry density to a higher extend (i.e., 1.15 g/cm^3^) which is not preferable for bioengineered structures (stability), and hence not considered in the present study. Three numbers of individual grass with equal numbers of leaves were transplanted in each column by digging small holes on soil surface. The columns were kept under a transparent-roofed house where samples were protected from natural precipitation however accessed to the sun light and air circulation i.e., maintained with natural environmental condition (Fig. 2). The air temperature, relative humidity and solar radiation around the house were monitored using a microclimate monitoring system (ATMOS 14^52^). The average temperature, relative humidity and solar radiation during the study period were found to be 23 ± 4 °C, 81 ± 9% and 124 ± 38 W/m^2^ respectively. The free or potential water evaporation was measured by the weight difference technique and found to be 4.4 ± 1.9 mL/h. Equal amount of water (250 ml) was irrigated 1-day interval in all the columns for maintaining moisture content at around field capacity or near saturated in the root zone. Water irrigation was continued till the vegetation near completely covered the soil surface i.e., fully grown over the columns. After completion of vegetation growth, the soil columns were saturated with water using a controlled head mariotte bottle with a flow rate of 100 ml/min until the suction read by the TEROS-21^53^ sensor down to the 9 kPa (minimum value). The completion of the saturation was ensured by the constant suction of around 9 kPa. Finally, the saturated columns were undergone continuous drying and the sensors (TEROS-21 and EC-5) were connected to the EM-50 data loggers for continuous monitoring of the suction and moisture content at an interval of 10 min. It is to be noted that the sensors were pre calibrated for the specific soil and biochar types tested. The growth and health parameters of the vegetation were monitored both in the growing and dying stage for almost 10-month.

The measured suction and water content data were fitted to van Genuchten^54^ model by least square method using RETC software^55^. The plant available water content (PAWC) was calculated from the field capacity (FC) and permanent wilting point (PWP).

#### Quantification of vegetation growth

**Figure 3:**
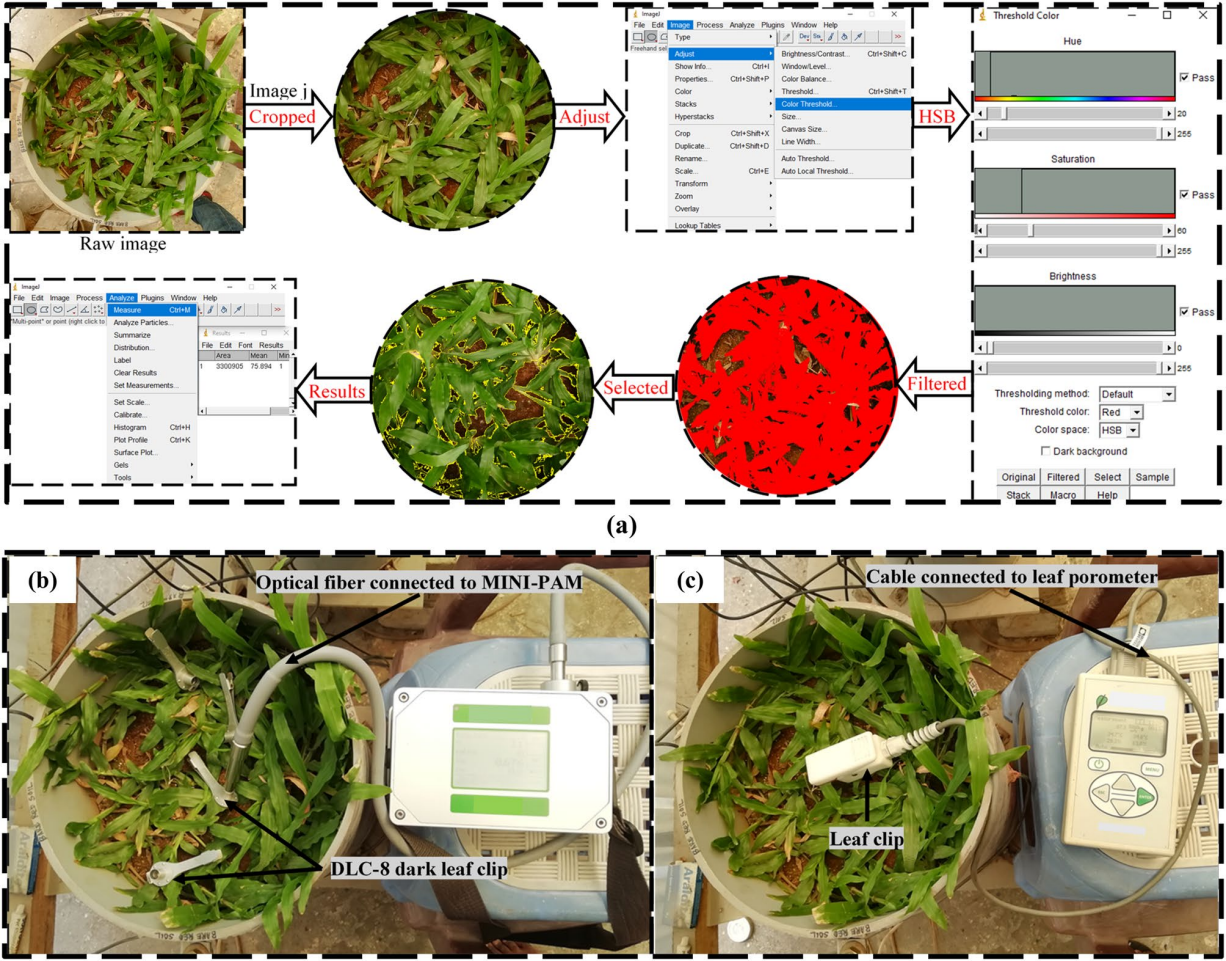
(**a**) steps for determining VD by image analysis technique and determination of, (**b**) photosynthetic yield and (**c**) stomatal conductance.

The growth of the vegetation was quantified by vegetation density (VD), leaves count, shoot mass and root mass density (RMD). VD (m^2^/m^2^) on a surface is defined as the ratio of area covered by the vegetation (A_v_) and the total soil surface area (A_s_) considered^56^. VD was measured from the captured images of the column surface by image analysis technique as highlighted in Fig. 3a. Images were captured using a digital camera (Canon, EOS 600D) in 2 days interval and from a same elevation (400 mm, Fig. 2b) of the soil surface to minimize any observational error. To start the image processing, the camera captured images were loaded into Image J, an open sourced Java based image processing software^56^ and cropped to only account the soil surface area. The pixel area of the cropped images i.e., the total soil surface area, *A*_*s*_ was counted. In the next step, the cropped images were adjusted to color threshold using HSB (Hue, Saturation and Brightness) color space and the corresponding pixel area (*A*_*v*_, red) of the vegetation was counted. Finally, the VD was calculated as follows:

1 $$VD(\%)=\frac{Av}{As}\times 100$$

The dry biomass of the shoot and roots after complete wilting of the grass was determined according to the method described in Abbas et al.^57^. The above ground biomass or shoot of the grass was cut and washed with distilled water. The roots along the depth were collected after taking out the soil and washed with 1% HCL and distilled water. The dry mass of roots was measured for each 2 cm depth and the total roots mass per unit volume of soil is presented as RMD. Finally, the total dry weights (oven-dried at 60 °C) of the shoot and roots were recorded after constant drying i.e., negligible to no change in the mass of the samples.

#### Measurement of stomatal conductance and photosynthetic yield

Stomatal conductance (SC) and photosynthetic yield (PY) were measured using leaf porometer (Meter Inc., USA) and MINI-PAM-II (Heinz Walz Gmbh, Effeltrich, Germany) instrument as shown in Fig. 3b,c. Leaf porometer gives the SC of leaves based on the relative humidity and temperature measured by the sensors placed across the leaf in series. To start the measurement of SC, the desiccant chamber in the porometer was filled with fresh desiccant and then calibrated with filter paper and deionized water for reliable measurement as suggested by the manufacturer. With the porometer calibrated, the leaf was placed in the leaf clip and the measurements were taken. The leaves in the leaf clip were placed carefully in order to minimise the leaves disturbances. Conductance was measured at the bottom of the grass leaf (abaxial conductance) due to the presence of higher numbers of stomata in the bottom surface of the grass leaves as the bottom surface is protected from the direct sun light. Large numbers of leaves are available in a single column and measuring SC in all is time consuming. Further, SC varies with the leaf age, time of measurement (diurnal variation) and location of the leaves i.e., under light or shade^9^. Therefore, four numbers of leaves with relatively same age and at different location (both shade and light) were selected. Measurements were taken within 10 A.M. to 1 P.M. of the day to avoid the effect of diurnal variation. Taking of measurement in the same selected leaves for the entire monitoring period was not possible due to the gradual wilting of grass leaves with age. Therefore, different fresh leaves for measurement were selected after wilting of the previous selected leaves. However, at the end of monitoring, when all the leaves were wilted, the measurements were taken in wilted leaves only.

The MINI-PAM could measure many photosynthetic parameters based on the chlorophyll fluorescence technique; however, in the present study, maximum yield (F_v_/F_m_), effective yield (Y(II)) and the photosynthetic efficiency were measured in dark actinic condition. The F_v_/F_m_ is the maximum light absorption and utilization (in photosynthesis) capacity of the leaves and occurs just after dark adaptation. Y(II) is the photochemical utilization of absorbed light by leaves under light condition. The photosynthetic efficiency was determined by measuring the light curve, which is the relationship between electron transfer rate (ETR) and light intensity expressed as photosynthetic active radiation (PAR). For measuring the photosynthetic parameters, the selected leaves (i.e., the same considered for SC measurement) were clamped using DLC-8 dark leaf clip with a moveable shuttle and allowed to adapt dark for 15 min. The clips were placed carefully to reduce the leaves disturbances as minimum as possible. With the leaves dark adapted, the optical fiber connected to the MINI-PAM was positioned in the clip and the readings were recorded after opening the shuttle (Fig. 3b). Measurements were taken on adaxial (top surface) leaf side as in the top surface presents higher density of chlorophyll due to direct exposure of sun light. The dark period of 15 min was selected based on the trial measurements where no significant difference in readings of dark adapted between 10 to 30 min was observed and it was also adapted by the past researchers^58,59^. All methods were carried out in accordance with relevant national and international guidelines.

## Results and discussion

### Physicochemical properties of the soil, biochar and BAS

**Figure 4:**
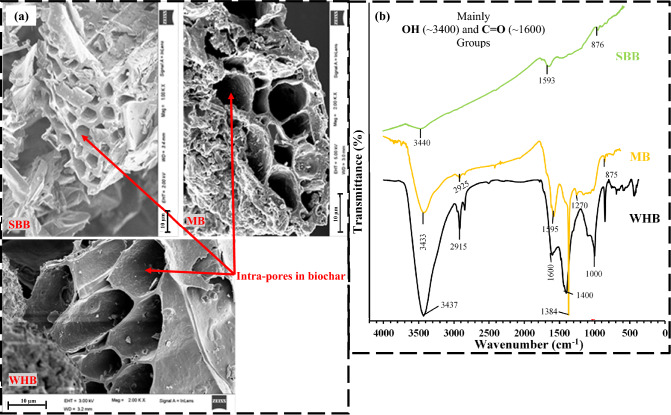
(**a**) FE-SEM images (at 2KX) and (**b**) FTIR spectra of different biochar tested.

The physicochemical properties of the soil, biochar and BAS are presented in Tables 1 and 2. The biochar exhibited considerably higher carbon (C) content and the MB showed the highest C among the biochar. The Ash content (AC) was observed to be very high in WHB and lowest in SBB (Table 1). The high AC could be beneficial for vegetation growth due to the added fertilizer effects. The SBB consists of Tridymite as major mineral while in the WHB and MB present calcite mineral. The SSA presented in Table 1 found to be higher in SBB due to the presence of large number smaller size and thick-walled pores in SBB compared to other biochar tested as seen from the FESEM images (Fig. 4a). The SSA is a crucial indicator of water and nutrient adsorption ability of biochar that could determine their applications in vegetated soil based on the water use efficiency and nutrient uptake. The FTIR analysis (Fig. 4b) revealed the presence of mainly hydrophilic –OH (web number 3433, 3437, 3440) and C=O (~ 1600, ionisable carboxyl group) functional groups in all the biochar^60,61^.

**Table 1:** Properties of the different biochar used.

Properties	SBB	WHB	MB
**Elemental composition (%)**			
Carbon (C)	65	53.39	71.5
Oxygen (O)	30	42.80	–
Hydrogen (H)	3.9	1.99	–
Nitrogen (N)	0.16	1.82	0.19
**Molar ratios**			
H:C	0.72	0.45	–
C:N	542	34.22	382
O:C	0.35	0.6	–

Major mineral (XRD)	Tridymite (SiO_2_)	Calcite (CaCO_3_)	Calcite (CaCO_3_)
Yield (%)	25	33	–
Ash content (%)	1	11	1.9
Volatile matter (%)	22.6	30	–
BET, SSA (m^2^/g)	41	30.15	21.54

**Table 2:** Physicochemical properties of the BAS.

Properties	Biochar type	Bare	5% BAS	10% BAS	15% BAS	Biochar
Specific gravity (–)	MB	2.68 ± 0.10	2.59 ± 0.04	2.47 ± 0.09	2.30 ± 0.16	1.44 ± 0.10
	WHB	2.68 ± 0.10	2.51 ± 0.05	2.42 ± 0.03	2.28 ± 0.04	0.80 ± 0.05
	SBB	2.68 ± 0.10	2.53 ± 0.05	2.44 ± 0.02	2.29 ± 0.04	1.11 ± 0.20
Water absorption capacity (%)	MB	36 ± 1.4	51 ± 3.5	58 ± 2.5	72 ± 3	156 ± 11
	WHB	36 ± 1.4	46 ± 3	60 ± 5	104 ± 6	564 ± 16
	SBB	36 ± 1.4	59 ± 1.5	79 ± 5	98 ± 0.5	633 ± 3.4
Liquid limit (%)	MB	49.9 ± 0.3	51.1 ± 0.4	52.7 ± 0.4	55.4 ± 0.4	–
	WHB	49.9 ± 0.3	53.5 ± 0.5	61 ± 0.6	72.6 ± 0.8	
	SBB	49.9 ± 0.3	55.7 ± 0.4	67.9 ± 0.5	78.9 ± 0.4	
Plastic limit (%)	MB	29.9 ± 1.1	31.8 ± 0.5	36.3 ± 0.1	37.7 ± 0.7	–
	WHB	29.9 ± 1.1	33.9 ± 0.4	42.5 ± 0.6	51.4 ± 0.4	
	SBB	29.9 ± 1.1	35.6 ± 1	40.5 ± 0.6	48.4 ± 0.5	
Shrinkage limit (%)	MB	27.3 ± 0.2	30.4 ± 0.70	32.5 ± 0.31	35.40 ± 0.40	–
	WHB	27.3 ± 0.2	33.4 ± 0.8	45.2 ± 0.9	63.3 ± 1	
	SBB	27.3 ± 0.2	37.5 ± 1.1	51.6 ± 0.4	68.7 ± 0.2	
MDD (kg/m^3^)	MB	1720	1600	1470	1430	449
	WHB	1720	1640	1570	1490	–
	SBB	1720	1622	1375	1177	–
OMC (%)	MB	17	17.9	19	20.5	–
	WHB	17	18.5	22	24	
	SBB	17	19	28	35	
pH (–)	MB	4.31 ± 0.05	5.52 ± 0.13	6.6 ± 0.06	7 ± 0.06	7.9 ± 0.3
	WHB	4.31 ± 0.05	6.7 ± 0.03	7.2 ± 0.05	7.63 ± 0.03	9 ± 0.32
	SBB	4.31 ± 0.05	6.13 ± 0.04	7.09 ± 0.01	7.4 ± 0.01	9.6 ± 0.01
EC (dSm^−1^)	MB	0.04 ± 0.003	0.12 ± 0.013	0.2 ± 0.02	0.34 ± 0.01	1.5 ± 0.01
	WHB	0.04 ± 0.003	0.12 ± 0.01	0.24 ± 0.02	0.37 ± 0.006	1.9 ± 0.02
	SBB	0.04 ± 0.003	0.12 ± 0.008	0.34 ± 0.07	0.46 ± 0.034	3.2 ± 0.028
CEC (meq/100 g)	MB	2.4	3	2.8	3.5	10
	WHB	2.4	9.5	12.8	15	27
	SBB	2.4	12.2	15.6	20	44

Value in table represents the average value ± standard deviation.

*CEC* cation exchange capacity, *EC* electric conductivity.

The soil tested was classified as silty sand, SM (USCS)^62^. The Atterberg limits (i.e., liquid limit, plastic limit and shrinkage limit) and water absorption capacity of the soil increased after amendment of all three types biochar (Table 2). The specific gravity (SG) and dry density of the soil decreased when biochar of all three types were amended (Table 2). The internally porous structure and lower SG of biochar compared to soil reduced the overall or composite SG and compressibility during compaction thereby lowered the dry density^34,63^. The pH, EC and CEC of the soil were observed to be increased after biochar amendment. The biochar exhibited higher pH (7.9–9.6) relative to the soil that have changed soil pH from acidic to neutral state. The higher CEC of biochar compared to soil attributing the presence of O– containing functional groups enhanced the soil CEC after amendment. The growth and development of vegetation needs soil media to be in pH neutral and have higher nutrient retention capacity (high CEC) which can be offered by BAS^64^. Further, variation in soil physicochemical properties was also observed with respect to biochar types (Table 2).

### SWRC of biochar-amended vegetated soil

**Figure 5:**
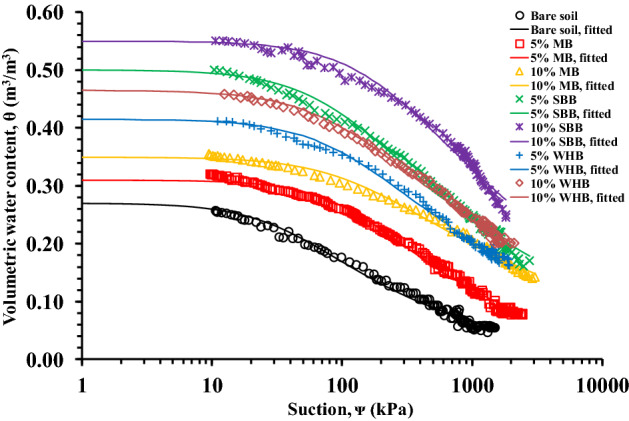
SWRCs of different biochar-amended vegetated soil.

Figure 5 shows the SWRC of the 5% and 10% MB, SBB and WHB-amended vegetated soil. The fitting coefficients of the SWRC are presented in Table 3 where the R^2^ values are observed to be in the range of 0.98–0.99. The saturated water content (*θ*_*s*_) of the soil was observed to be increased in the range of 15–104% after amendment of different types biochar. The higher porosity of the soil after biochar amendment as evident from the lower dry density of BAS (Table 2), and the –OH and C=O functional groups present in biochar surface (Fig. 4b) which are polar in nature^60^ attract and held higher volume of water in BAS relative to bare soil thus, the higher *θ*_*s*_ in BAS. The internally porous biochar as seen from the FESEM images (Fig. 4a) and WAC (Table 2), and restructure of inter-pores between particles of different size soil and biochar led to the higher porosity in BAS. The SBB-amended soil exhibited the highest *θ*_*s*_ among the biochar types tested. The higher porosity of SBB-amended soil compared to other BAS as evident from the lowest dry density and highest WAC (Table 2) attributed to the highest *θ*_*s*_. The increase of soil porosity after biochar amendment was also reported by the past researchers^34,35,65^.

**Table 3:** Fitting parameters of SWRCs fitted using van Genuchten^54^ model.

Biochar type	BC (%)	θ_s_ (m^3^/m^3^)	θ_r_ (m^3^/m^3^)	α (m^−1^)			AEV (kPa)	n (–)			M (–)	R^2^	θ_1500_ (m^3^/m^3^)	PAWC (%)
				Value	SE	t		Value	SE	t				
Bare	0	0.27	0	0.24	0.01	24	18	1.43	0.01	175	0.30	0.99	0.055	17
MB	5	0.31	0	0.072	0.002	38	58	1.46	0.01	201	0.32	0.99	0.10	20.7
	10	0.35	0	0.071	0.004	17	62	1.28	0.01	176	0.22	0.99	0.18	17.3
WHB	5	0.415	0	0.088	0.004	24	46	1.32	0.01	214	0.24	0.99	0.18	22.9
	10	0.465	0	0.097	0.004	26	42	1.277	0.005	259	0.22	0.99	0.22	24.4
SBB	5	0.50	0	0.101	0.004	24	42	1.31	0.01	233	0.23	0.99	0.22	26.4
	10	0.55	0	0.044	0.003	15	90	1.32	0.01	100	0.24	0.98	0.30	25.7

*BC* biochar content, *AEV* air entry value, *PAWC* plant available water content, *SE* standard error, *t* t value.

The air entry value (AEV) of the soil was observed to be increased in the range of 133–400% after amendment of different types biochar. The AEV is a unique suction value beyond which the increased soil suction causes the entrapment of air in soil and it is related to the SWRC shape parameter *α*. The AEV is dependent on the soil pore size therefore, the higher AEV in BAS indicates the smaller size pores and strong held of water in pores. The amendment of biochar made pore size distribution of the soil tortuous or complex with smaller size pores^66^ that have extended the resistance to water evaporation and hence the higher AEV. The SBB-amended soil exhibited the highest AEV among the tested biochar attributed to the smaller size pores in SBB relative to other biochar (Fig. 4a). The shape parameter *n* which defines the slope of the transition zone was observed to be decreased in the range of 7–11% after biochar amendment. Similar to AEV, the *n* is also related to the pore size distribution of soil thus, the lower *n* in BAS implies the reduced pore size relative to pores size in bare soil as also evident from the higher AEV. The reorganization of soil pores into smaller and larger sizes after biochar amendment as observed in the present study was also reported by the previous researchers Sun et al.^66^ and Wong et al.^32^.

**Figure 6:**
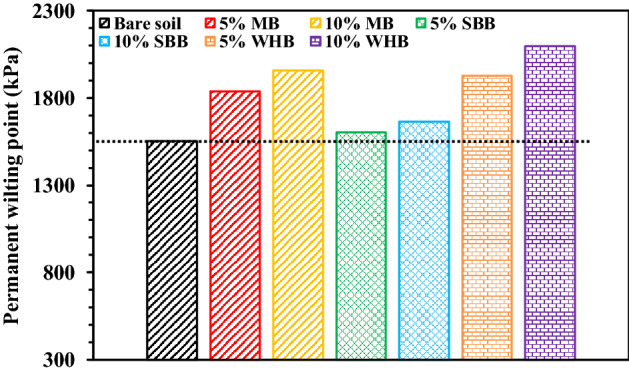
Variation of permanent wilting point in different biochar-amended vegetated soil.

The fitting of coefficient residual water content (*θ*_*r*_) yield magnitude as zero and the measured suction was not large enough to the residual suction. Therefore, the *θ*_*1500*_ i.e., the water content corresponds to suction of 1500 kPa was evaluated as adopted in Hussain et al.^16^ for quantifying and comparing the biochar effect at higher suction. The *θ*_*1500*_ was found to be increased in the range of 82–445% after biochar amendment. The smaller size pores in BAS which may be intra-pores or inter-pores and can hold water tightly at larger suction due to their strong capillary action led to the higher *θ*_*1500*_ in BAS^8^. The SBB-amended soil exhibited the highest *θ*_*1500*_ among the biochar types tested attributed to their smallest intra-pores size as seen from the FESEM images (Fig. 4a). The PAWC, which was evaluated from the measured PWP found to be increased in the range of 22–55% after biochar amendment. Relatively higher increase in PAWC was observed with SBB compared to other biochar tested (Table 3). The variation of PWP in different BAS is presented in Fig. 6 and the PWP is found to be higher (3–35%) in BAS compared to the bare soil, indicating the survivability of vegetation at a larger drought stress in BAS relative to bare soil. Thus, the improved water retention in biochar-amended vegetated soil due to the altered pore structure and action of functional groups is an encouraging sign for the implementation of BAS in bioengineered structures.

### Effect of biochar on vegetation growth and its relation with soil suction

**Figure 7:**
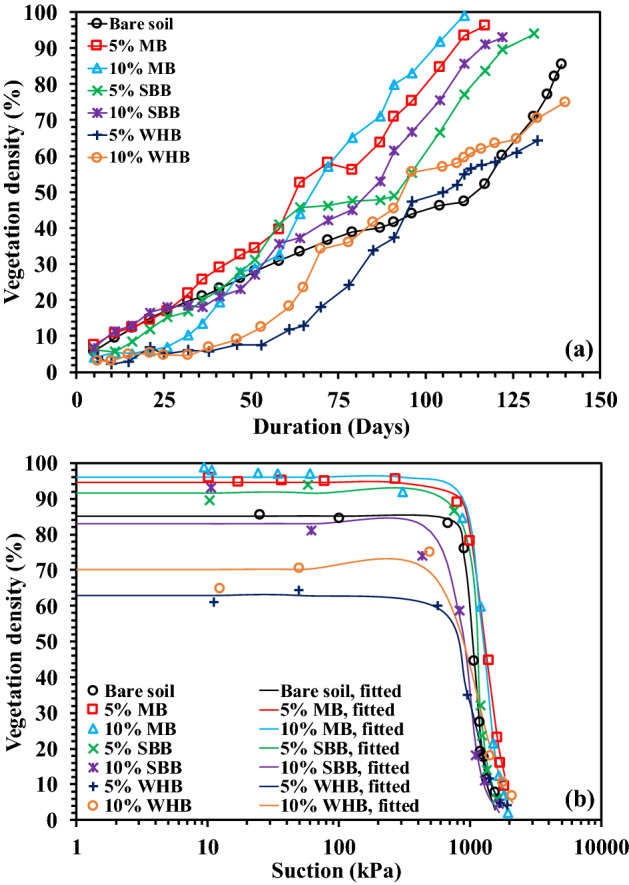
Variation of vegetation density with (**a**) time and (**b**) suction in different BAS.

**Figure 8:**
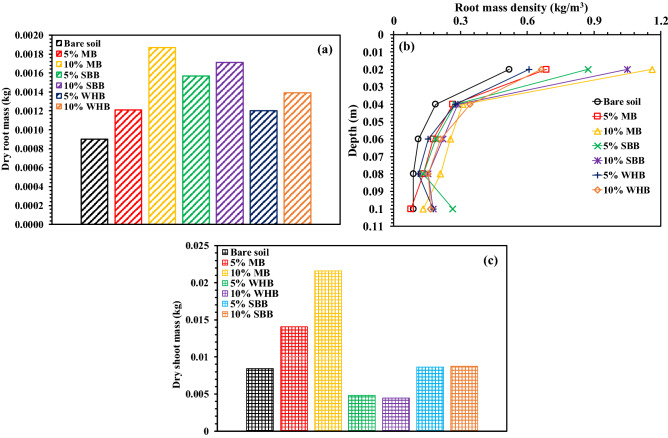
Variation of (**a**) dry root mass, (**b**) root mass density and (**c**) dry shoot mass in different BAS.

Figure 7a,b shows the variation of vegetation density (VD) with time and suction in 5% and 10% MB, WHB and SBB-amended soil. The day zero in Fig. 7a indicates the day of transplantation of the grass. The VD was observed to be increased with time and reached the maximum or peak at which the vegetation near completely covered the column surface. The final VD was observed to be higher in BAS relative to bare soil, except the WHB-amended soil that have exhibited relatively lower VD compared to bare soil. The amendment of biochar decreased dry density (Table 2), and increased water retention capacity i.e., higher plant available water (Fig. 5, Table 3), pH and EC (Table 2) of the soil which have enhanced the growth of roots and vegetation or VD. The total dry root mass presented in Fig. 8a was found to be higher in BAS compared to the bare soil. The higher roots mass in BAS is beneficial as these could provide more anchorage to the top soil against erosion and failure. The decrease of soil dry density or increase of porosity facilitates the roots to easily penetrate through soil without resistance. Further, the root mass density (RMD, Fig. 8b) showed a gradual decrease along the depth in both bare and BAS. The grass species tested in the present study had fibrous root system due to which most of the roots were grown at the top surface. In addition, the higher soil confinement along the depth restricted the root growth at the deeper depth. The shoot and root mass of a grass (*cynodon dactylon*) was found to be increased after 2 years of monitoring due to amendment of peanut shell biochar^38^. Similar higher growth of root and shoot were also reported by Ng et al.^36^ for *schefflera arboricola* grown in peanut shell and wood biochar-amended soil.

The higher or near neutral pH of BAS offers a suitable medium for the enzymatic and microbial activity by enhancing the microbial diversity and community structure in the root zone that helps in the efficient cycling of nutrient for plant use. Previous studies on soil-biochar-microbial interaction found an enhanced microbial diversity and community structure along with improved physicochemical properties in soil after biochar amendment^23,67,68^. Soil enzymatic activities that controls the soil nutrient cycling ability were found to be increased after biochar amendment^36,69^. The MB-amended soil exhibited the highest VD among the biochar types tested. Although, the SBB and WHB-amended soil exhibited a higher water retention and lower dry density compared to MB-amended soil, the higher pH and nutrient content in MB played the role in enhancing the vegetation growth or VD. The results of dry shoot mass (Fig. 8c) showed a higher dry shoot mass in MB-amended soil relative to bare soil. However, the amendment of SBB showed no significant change, and the amendment of WHB exhibited a decrease in dry shoot biomass relative to bare soil.

The measured VD in different BAS was plotted against suction (Fig. 7b) and the plots were fitted to the correlation proposed by Gadi et al.^9^ as given below:

2 $$VD\left(\Psi \right)={VD}_{min}+\frac{{VD}_{max}-{VD}_{min}}{[1+{{\left(a\left(\Psi \right)\right)}^{n}]}^{1-\frac{1}{n}}}$$

where *VD* (*Ψ*) is the vegetation density as function of suction (m^2^/m^2^), ${VD}_{max}$ is the maximum vegetation density (m^2^/m^2^), ${VD}_{min}$ is the minimum vegetation density (m^2^/m^2^), $\alpha$ is the fitting parameter related to the suction at which VD start decreasing or wilting starts ($\alpha >$ 0) and *n* is the fitting coefficient associated with the curvature or the slope of the curve (n > 1). The fitting parameters are presented in Table 4. The R^2^ value was observed to be in the range of 0.97–0.99. The suction at which the wilting start is termed as *ψ*_*w*_ and graphically measured from the curves. It could be observed from Fig. 7b that the increase of soil suction due to evapotranspiration caused no change in the VD up to a suction equivalent to *ψ*_*w*_ and further increase of suction beyond *ψ*_*w*_ decreased the VD and reached the minimum at PWP. The biochar amendment showed no consistent effect on *ψ*_*w*_ except the MB-amended soil, where *ψ*_*w*_ increased relative to the bare soil. The VD gradually decreased in BAS as observed from the smaller *n* value (Table 4) whereas a steep decrease of VD (larger *n* value) could be observed in bare soil. The PWP refers to suction (Fig. 6) where vegetation was completely wilted captured based on the negligible value (reading) of plant health parameters (i.e., SC and PY). The PWP is significant for vegetation growth as the approaching of drought stress near PWP could lead to the plant death. Further, biochar amendment changes pore structure (suction) and hence the water retention characteristics of soil therefore, the PWP is expected to change which necessitates the determination of PWP. Therefore, the biochar amendment increased the vegetation growth and slowed down the wilting. In addition, the MB-amended soil showed the highest vegetation growth and slowest wilting among the biochar types tested under the influence of drought or increased suction which will be beneficial for the bioengineered structures.

**Table 4:** Fitting parameters of plant parameter vs suction curve obtained after fitting the correlation proposed by Gadi et al.^9^.

Plant parameter	Fitting parameters	Bare	MB		WHB		SBB	
			5%	10%	5%	10%	5%	10%
VD	VD_max_ (%)	85	94.5	96	63	70	91.5	83
	VD_min_ (%)	0	0	0	0	0	0	0
	α (kPa^−1^)	0.001	0.001	0.001	0.001	0.001	0.001	0.001
	Ψ_W_ (kPa)	800	950	900	700	650	1000	730
	n (–)	10.1	6.4	7.6	5.1	5.1	8.1	7
	m (–)	0.9	0.8	0.9	0.8	0.8	0.9	0.9
	R^2^ value	0.99	0.99	0.99	0.99	0.99	0.99	0.98
SC	SC_max_ (mmol/m^2^ s)	254	193	242	198	218	211	183
	SC_min_ (mmol/m^2^ s)	24	24	24	24	24	24	24
	α (kPa^−1^)	0.002	0.001	0.003	0.004	0.005	0.006	0.004
	Ψ_SCD_ (kPa)	220	350	150	55	90	72	85
	n (–)	2.31	2.7	1.81	1.95	1.83	2.42	2.35
	m (–)	0.57	0.63	0.45	0.49	0.45	0.59	0.57
	R^2^ value	0.95	0.94	0.93	0.97	0.98	0.99	0.98
F_v_/F_m_	(F_v_/F_m_)_max_ (–)	0.77	0.76	.75	0.72	0.73	0.75	0.75
	(F_v_/F_m_)_min_ (–)	0	0	0	0	0	0	0
	α (kPa^−1^)	0.001	0.001	0.001	0.001	0.001	0.001	0.001
	Ψ_(Fv/Fm)D_ (kPa)	1050	1550	1600	1250	1070	1100	1000
	n (–)	7.4	19.3	15	10.7	5.1	39.7	31.4
	m (–)	0.86	0.95	0.93	0.91	0.80	0.97	0.97
	R^2^ value	0.94	0.99	0.99	0.99	0.99	1	1
PY (II)	PY(II)_max_ (–)	0.74	0.73	0.72	0.70	0.71	0.72	0.71
	PY(II)_min_ (–)	0	0	0	0	0	0	0
	α (kPa^−1^)	0.001	0.001	0.001	0.001	0.001	0.001	0.001
	Ψ_(PY(II))D_ (kPa)	1050	1550	1600	1250	1070	1100	1000
	n (–)	7.9	19.3	15.3	10.3	4.8	45.9	15.9
	m (–)	0.87	0.95	0.93	0.90	0.79	0.98	0.94
	R^2^ value	0.94	1	0.98	0.99	0.99	1	1

### Variation of SC and PY with suction in different biochar-amended soil

**Figure 9:**
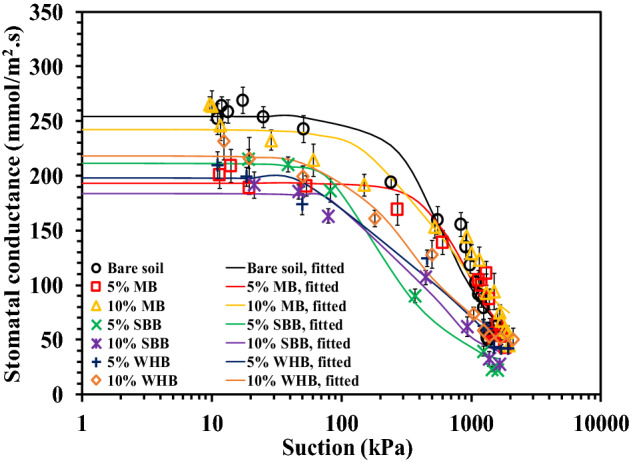
Variation of SC with suction in different BAS.

Figure 9 presents the variation of SC with suction in different BAS. The measured SC versus suction was found to fit better (R^2^ value of 0.93–0.99) the correlation proposed by Gadi et al.^9^ given below

3 $$SC (\Psi )={SC}_{min}+\frac{{SC}_{max}-{SC}_{min}}{[1+{{(a\left(\Psi \right))}^{n}]}^{1-\frac{1}{n}}}$$

where *SC* (*Ψ*) is the stomatal conductance as a function of suction (mmol m^−2^ s^−1^/mmol m^−2^ s^−1^), *SC*_*max*_ is the maximum stomatal conductance (mmol m^−2^ s^−1^/mmol m^−2^ s^−1^), *SC*_*min*_ is the minimum stomatal conductance (mmol m^−2^ s^−1^/mmol m^−2^ s^−1^), α is the shape parameter related to the suction corresponding to peak SC ($\alpha >$ 0) and *n* is the fitting coefficient associated with the curvature or the slope of the curve (n > 1). The suction at which the SC start decreasing is termed as *Ψ*_*SCD*_ and measured graphically from the curve. The fitting parameters are presented in Table 4. At relatively lower suction range or suction lower than *Ψ*_*SCD*_, the peak SC was observed to be unchanged with suction in all bare and BAS. The lower soil suction where abundant water is available for the plant to uptake facilitates the maximum transpiration or SC from the plants^36^. As presented in Table 4, the maximum SC (*SC*_*max*_) was observed to be lower in BAS compared to the bare soil. This could be attributed to the maximum utilisation of root up taken water in the growth of vegetation as the VD and roots mass were recorded to be higher in BAS and hence lower loss (transpiration) through stomata.

Early decrease of SC as suggested by the lower *Ψ*_*SCD*_ value (Table 4) could be observed in BAS relative to bare soil. However, further increase of suction beyond *Ψ*_*SCD*_ gradually decreased the SC in BAS as quantified by lower *n* value in BAS compared to the bare soil of large *n* value (Table 4). With the increase of suction or decrease of water content in root zone, the vegetation gradually modifies its physiological activity like partial to complete closure of stomata by releasing plant hormone^70^ to curb down the transpiration and meet the water deficiency in soil. This modification of plant physiological activity with increased suction sometime represented as transpiration reduction function^7^. The early decrease of SC in BAS could be attributed to the partial closure of stomata due to the release of abscisic acid (ABA, plant hormone). The amendment of biochar increases the release of ABA for the intension of improving the roots growth and the resistance against drought stress and pathogen^71,72^. The *SC*_*min*_ was fixed at 24 mmol m^−2^ s^−1^ since the leaf porometer showed a minimum SC reading in the range 20–25 mmol m^−2^ s^−1^ in completely wilted leaves. Ideally, in completely wilted leaves, zero to negligible SC reading is expected, however leaf porometer evaluates the SC based on the measured temperature and relative humidity, and a slight change in these parameter could yield a large SC value.

**Figure 10:**
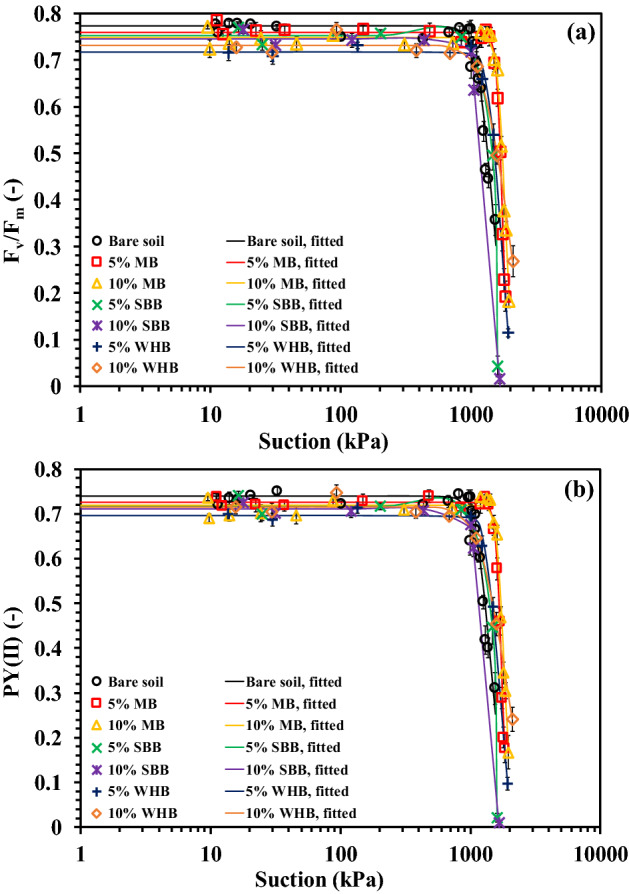
Variation of (**a**) F_v_/F_m_ and (**b**) PY (II) with suction in different BAS.

Figure 10a,b highlights the variation of maximum (F_v_/F_m_) and effective (PY(II)) photosynthetic yield (PY) with suction in different BAS. The PY versus suction plot followed the similar pattern of SC versus suction. The peak value of (F_v_/F_m_) and PY(II) were observed to be above 0.7 on a measuring scale of 0–0.84 which indicates the healthy condition of the leaves or the vegetation. The peak PY value of around 0.7 was also reported for grass species in literature^50^. To correlate the PY with suction and to see the effect of biochar amendment, the measured PY data was fitted to the correlation given below

4 $$\frac{{F}_{v}}{{F}_{m}} (\Psi )={\left(\frac{{F}_{v}}{{F}_{m}}\right)}_{min}+\frac{{\left(\frac{{F}_{v}}{{F}_{m}}\right)}_{max}-{\left(\frac{{F}_{v}}{{F}_{m}}\right)}_{min}}{[1+{{(a\left(\Psi \right))}^{n}]}^{1-\frac{1}{n}}}$$

5 $$PY(II) (\Psi )={PY(II)}_{min}+\frac{{PY(II)}_{max}-{PY(II)}_{min}}{[1+{{(a\left(\Psi \right))}^{n}]}^{1-\frac{1}{n}}}$$

where *F*_*v*_*/F*_*m*_ (*Ψ*) and *PY(II)* (*Ψ*) are the maximum and effective photosynthetic yield as a function of suction (–), (F_v_/F_m_)_max_ and PY(II)_max_ are the peak value of the (F_v_/F_m_) and PY(II) (–), (F_v_/F_m_)_min_ and PY(II)_min_ are the minimum value of the (F_v_/F_m_) and PY(II) (–), α is the shape parameter related to the suction corresponding to peak (F_v_/F_m_) and PY(II) ($\alpha >$ 0) and *n* is the fitting coefficient associated with the curvature or the slope of the curve (n > 1). The fitting parameters are presented in Table 4. The R^2^ value (0.94–1) indicates the best fit of the correlation. The suction where the (F_v_/F_m_) and PY(II) start decreasing is termed as *Ψ*_*(Fv/Fm)D*_ and *Ψ*_*(PY(II))D*_, and measured graphically from the curve. There was no comparable variation observed in the (F_v_/F_m_)_max_ and PY(II)_max_ after biochar amendment. The (F_v_/F_m_)_max_ and PY(II)_max_ were observed to be unchanged for suction in the range of 1000–1600 kPa i.e., upto *Ψ*_*(Fv/Fm)D*_ and *Ψ*_*(PY(II))D*_. The BAS exhibited higher *Ψ*_*(Fv/Fm)D*_ and *Ψ*_*(PY(II))D*_ compared to the bare soil (Table 4), indicating the complete photosynthetic activity even at higher suction in BAS. The higher plant available water due to smaller pores size and the delayed wilting (higher *Ψ*_*W*_, Table 4) in BAS relative to bare soil allowed the complete photosynthetic activity or yield at a comparatively higher suction i.e., higher *Ψ*_*(Fv/Fm)D*_ and *Ψ*_*(PY(II))D*_. The increase of suction beyond *Ψ*_*(Fv/Fm)D*_ and *Ψ*_*(PY(II))D*_ decreased the PY and ceased at PWP, where vegetation fractionally lost the photosynthetic activity due to the permanent closure of reaction centre in the chloroplast. A steep or abrupt decrease of the (F_v_/F_m_) and PY(II) as quantified by the large *n* value (Table 4) could be observed in BAS relative to bare soil. The increase of soil suction or drought stress beyond *Ψ*_*(Fv/Fm)D*_ and *Ψ*_*(PY(II))D*_ led to the water deficiency and wilting in the plants which in turn destroyed the reaction centre or photo receptor in photosystem-II of chloroplast thus, the lower absorption of photon or lower PY^50,73^. Considering the effect of biochar types on PY, the MB-amended soil exhibited the highest *Ψ*_*(Fv/Fm)D*_ and *Ψ*_*(PY(II))D*_ among the biochar types tested attributed to the highest plant growth (root mass and VD) and delayed wilting in MB-amended soil. Thus, the biochar amendment slowed down the decreasing rate of SC and the photosynthetic activity up to a large suction which indicates the healthier status of vegetation in BAS compared to bare soil and will be beneficial for the bioengineered structures.

## Conclusions

The following conclusion can be drawn from the study:

1. The biochar amendment increased the water retention of the soil by on an average 15–400% and improved the soil physicochemical properties i.e., increased the pH, CEC and WAC in the range of 6–189%, and decreased the dry density by 3–32%. The internally porous structure and distinct material characteristics of biochar that have increased porosity, and the hydrophilic functional groups present caused the improved water retention and other soil properties in BAS.
2. The vegetation growth was found to be improved i.e., increased the vegetation density by 8–13%, root mass by 33–108% and shoot mass by 4–157% after biochar amendment. Further, the wilting of the vegetation was slowed down to a higher suction i.e., the PWP increased by 3–35%. The decreased dry density and increased water retention or higher PAWC, pH, and CEC of the soil after biochar amendment attributed to the higher vegetation growth in BAS.
3. The amendment of biochar made the vegetation healthier i.e., decreased the SC for the intention of defence against drought stress and pathogen, and allowed the complete photosynthetic activity (peak PY) at a higher suction (1000–1600 kPa). The release of abscisic acid after biochar amendment which acts against the drought stress contributed to the decreased SC.
4. The different biochar types tested, the SBB-amended soil exhibited the higher improvement in water retention capacity while the MB-amended soil showed the higher vegetation growth and slower wilting or healthier status among the biochar types tested.

The present study showed that the vegetation does not even start wilting at suction 600 kPa, and the SC and PY remain unchanged at suction under 1000 kPa or starts decreasing at suction larger than 1000 kPa. However, the previous study measured suction only up to 90 kPa which was not able to capture the actual phenomenon. In addition, the soil properties and the vegetation growth in BAS found to be biochar types dependent. Therefore, the findings of the present study suggest the application of BAS in bioengineered structures which will have significant impact on the bioengineered or geotechnical structures. However, further field-scale studies are needed to better understand the soil-biochar-plant interaction.

## Data Availability

All data, models, and code generated or used during the study appear in the submitted article.

## Abbreviations

BAS: Biochar-amended soil
MB: Mesquite biochar
WHB: Water hyacinth biochar
SBB: Sugarcane bagasse biochar
SWRC: Soil water retention curve
θ_s_: Saturated volumetric water content
θ_r_: Residual volumetric water content
n: Shape parameter represents transition zone of the SWRC
AEV: Air entry value
CEC: Cation exchange capacity
SSA: Specific surface area
PWP: Permanent wilting point
PAWC: Plant available water content
SC: Stomatal conductance
PY: Photosynthetic yield
VD: Vegetation density
RMD: Root mass density
